# Nox4-IGF2 Axis Promotes Differentiation of Embryoid Body Cells Into Derivatives of the Three Embryonic Germ Layers

**DOI:** 10.1007/s12015-021-10303-x

**Published:** 2021-11-20

**Authors:** Jusong Kim, Jaewon Kim, Hee Jung Lim, Sanghyuk Lee, Yun Soo Bae, Jaesang Kim

**Affiliations:** 1 Department of Life Science, Ewha Womans University, 52 Ewhayeodae-gil, Seodaemun-gu, Seoul, 03760 Korea; 2 Ewha Research Center for Systems Biology, Seoul, 03760 Korea

**Keywords:** ROS, Nox4, ES cells, iPSCs, Embryoid body, IGF2

## Abstract

Reactive oxygen species (ROS) play important roles as second messengers in a wide array of cellular processes including differentiation of stem cells. We identified Nox4 as the major ROS-generating enzyme whose expression is induced during differentiation of embryoid body (EB) into cells of all three germ layers. The role of Nox4 was examined using induced pluripotent stem cells (iPSCs) generated from Nox4 knockout (Nox4^−/−^) mouse. Differentiation markers showed significantly reduced expression levels consistent with the importance of Nox4-generated ROS during this process. From transcriptomic analyses, we found insulin-like growth factor 2 (IGF2), a member of a gene family extensively involved in embryonic development, as one of the most down-regulated genes in Nox4^−/−^ cells. Indeed, addition of IGF2 to culture partly restored the differentiation competence of Nox4^−/−^ iPSCs. Our results reveal an important signaling axis mediated by ROS in control of crucial events during differentiation of pluripotent stem cells.

**Graphical Abstract:**

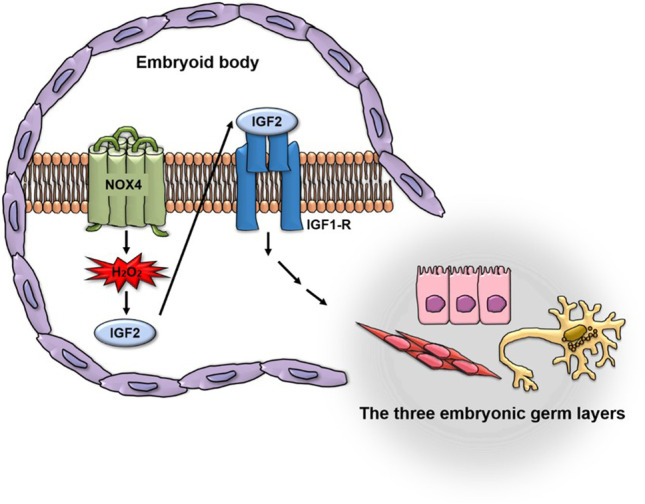

**Supplementary Information:**

The online version contains supplementary material available at 10.1007/s12015-021-10303-x.

## Introduction

The range of cellular processes ROS mediates is continuously expanding and includes those associated with cell signaling and homeostasis [1–3]. In fact, the role of ROS as second messengers is now widely accepted, and mechanisms involved in generation and removal of ROS are extensively studied. NADPH oxidase (Nox) enzyme gene family members in particular have been firmly established for their function in generating ROS in response to various signals such as extracellular growth factors and cytokines [4, 5]. Mouse possesses six members of this gene family (Nox1, Nox2, Nox3, Nox4, Duox1, and Duox2), while humans have one additional isozyme, Nox5. Their expression patterns are complex, overlapping, and dynamic. The exact assignment of their expression patterns is an important goal as isozyme-specific strategies for inhibition and activation are necessary to regulate these enzymes and thereby manipulate relevant cellular processes in precise manners. This goal is in particular relevant to developing therapeutic reagents for diverse pathologies including neurodegenerative diseases and cancer which are often consequences of inappropriate production of ROS and resulting oxidative stresses and cellular damages [6, 7].

Stem cells are important tools for cell-based regenerative therapies. ROS are known to regulate self-renewal and differentiation of various stem cells including pluripotent embryonic stem (ES) cells [6, 8, 9]. Nox2 and Nox4 have been most frequently reported to function as the source for ROS during stem cell differentiation [10–13]. Of note, most of the studies including those targeting ES cells and iPSCs are designed to find ways to promote unidirectional differentiation [14–17]. Typically, function of ROS or of a specific Nox isozyme was examined during differentiation of stem cells into one type of cell such as smooth muscle cells or cardiomyocytes [16, 17]. Also, in most cases, a monolayer culture was used to induce differentiation. At least for ES cells and iPSCs, this gives only a limited window to explore the role of ROS. ES and iPSCs can grow in a three-dimensional fashion and produce all somatic cellular derivatives of the three embryonic germ layers [18, 19]. Specifically, EB, grown in suspension, partly recapitulates the complexity of cell types from all three germ layers as well as cellular interactions seen during embryonic development. Therefore, this represents an opportunity to examine in vitro the role of ROS mediated signaling in cell differentiation and fate determination associated with early embryogenesis.

In this report, we examine for the first time the role of ROS during differentiation of EB cells into derivatives of all three germ layers. We show that only Nox4 is significantly induced during this process. Furthermore, using iPSCs derived from Nox4^−/−^ cells, we demonstrate that Nox4-dependent H_2_O_2_ generation is important for efficient differentiation of these cells. Finally, we present IGF2 as an important down-stream mediator of Nox4 signaling during differentiation of EB-derived cells.

## Materials and Methods

### Cell Culture

Mouse embryonic fibroblasts (MEF) were isolated from embryonic day (E) 13.5 CF-1 embryos. Briefly, after separating fetuses, the trunks were finely minced by passing through a syringe. The mixture of cells and small tissue masses was incubated with 0.05% trypsin-EDTA at 37℃ for 30 min with shaking. Digestion was terminated by adding FBS (Hyclone, Logan, Utah), and cells were resuspended in DMEM (Welgene, Daegu, Korea) supplemented with 10% FBS (Hyclone) and transferred to 150-mm culture dish. For preparation of MEF feeder cells, cells were typically cultured at 37℃ in atmosphere of 5% CO_2_ in DMEM (Welgene) supplemented with 10% FBS (Hyclone), 0.1mM MEM nonessential amino acid (Gibco, Carlsbad, CA), 2mM GlutaMAX (Gibco), 1% Penicillin/streptomycin (Gibco) and 0.1mM β-mercaptoethanol (Gibco). Mouse ES cells (ATCC; Manassas, VA, USA) and iPSCs were maintained on MEF feeder cells treated with 10 µg/ml mitomycin C (Sigma, St. Louis, MO) in DMEM supplemented with 15% FBS (Gibco), 0.1mM MEM nonessential amino acid (Gibco), 2mM GlutaMAX (Gibco), 1mM sodium pyruvate (Gibco), 1% Penicillin/streptomycin (Gibco), 0.1mM β-mercaptoethanol (Gibco) and 1000units/ml leukemia inhibitory factor (LIF; Millipore, Billerica, MA). For production of retroviruses, 293GPG packaging cells were maintained in DMEM containing 10% FBS (Hyclone), 0.1mM MEM nonessential amino acid (Gibco), 2mM GlutaMAX (Gibco), 1% penicillin/streptomycin (Gibco), 10mM HEPES buffer (Gibco), 8mM NaOH, 2 µg/ml puromycin (Sigma) and 300 µg/ml of geneticin (Gibco).

### Production of Retroviruses

The pMXs-IRES-Puro-based retroviral expression vectors for mouse Oct4, Soc2, Klf4, and c-Myc were obtained from Addgene (Cambridge, MA). Retroviral vectors were transfected to 293GPG packaging cells using Lipofectamine 2000 (Invitrogen, Carlsbad, CA) according to the manufacturer’s protocol. After transfection, 293GPG cells were selected using 2 µg/ml tetracycline (Sigma), 10 µg/ml puromycin (Sigma), and 300 µg/ml of geneticin (Gibco). For induction of viral production, selection agent-free medium was applied to 293GPG cells. Virus-containing supernatants were harvested, centrifuged and kept frozen until transduction of target cells. Further details are available upon request.

### Generation of iPSCs

MEF were seeded at 1.5 × 10^5^ cells per 35-mm dish the day before viral transduction. Viruses expressing mouse Oct4, Sox2, Klf4, and c-Myc were applied to cells twice over 2days at 1:1:1:1 ratio in the presence of 4 µg/ml polybrene. Subsequently, the cells were cultured in mouse ES medium for 4 days then in mouse ES medium containing 1.5ug/ml puromycin for selection with daily changes until colonies of iPSCs formed. Individual iPSC colonies were picked in 16 days to establish clonal cell lines.

### EB Formation and In Vitro Differentiation

Mouse ES or iPSC colonies were harvested using TrypLE (Gibco) and suspended in mouse ES medium without LIF. The single cells were cultured in hanging drop (3 × 10^3^/20µl) to form EB. After 3 days, aggregated cells were plated onto gelatin-coated coverslips in 12-well plates and cultured for differentiation. Diphenyleneiodonium chloride (DPI; Sigma) was applied at 250 nM from a 10mM stock solution in DMSO.

### Alkaline Phosphatase (AP) Staining

Cells were first washed with PBS, fixed with 4% paraformaldehyde for 10 min at room temperature and washed with distilled water. Cells were subsequently stained with 5-bromo-4-cloro-3-indolyl-phosphate/nitro blue tetrazolium color development substrate kit (Promega, Fitchburg, WI) according to the manufacturer’s protocol.

### Coomassie Blue Staining

Cells were washed with PBS, fixed with 4% paraformaldehyde for 10 min at room temperature and stained with 0.1% Coomassie Blue in 45% methanol and 10% acetic acids solution.

### Immunofluorescence

Cells were fixed with 4% paraformaldehyde for 10 min at room temperature, washed with PBS, and permeabilized in 0.5% Triton X-100 for 10 min. After blocking with PBS containing 1% bovine serum albumin for 1 h at room temperature, the cells were incubated with the following primary antibodies: anti-βIII-tubulin (Millipore), anti-smooth muscle actin (SMA; Dako, Glostrup, Denmark), anti-α-fetoprotein (Dako), anti-Oct4 (H-134; Santa Cruz Biotechnology, Santa Cruz, CA), anti-Sox2 (Millipore), anti-SSEA1 (Santa Cruz Biotechnology) antibodies overnight at 4℃. The secondary antibodies, Alexa Fluor 594-conjugated goat anti-mouse IgG (Life Technologies, Grand Island, NY), Alexa Fluor 594-conjugated goat anti-rabbit IgG (Life Technologies), Alexa Fluor 488-conjugated goat anti-mouse IgG (Life Technologies) or Alexa Fluor 488-conjugated goat anti-rabbit IgG (Life Technologies) were applied for 1 h at room temperature in the dark. Cells were counterstained with 4′,6-diamidino-2-phenylindole (DAPI) and examined with LSM 880 with Airyscan (Carl Zeiss, Jena, Germany).

### Measurement of ROS Levels

Cells were incubated with 5µM 2′,7′-Dichlorodihydrofluorescein diacetate(DCF-DA;Sigma) for 15 min at 37℃. The fluorescence intensity, reflecting intracellular ROS levels, was immediately measured using a FACS Calibur (San Jose, CA).

### RNA Extraction and Quantitative Real-Time PCR

Total RNA was extracted with TRIzol reagent (Invitrogen) following the manufacturer’s protocol. Synthesis of cDNA was carried out using 1 µg of RNA/20µl of reaction volume with QuantiTech Reverse Transcription Kit (Qiagen, Valencia, CA, USA) according to manufacturer’s protocol. KAPA Probe Fast qPCR master mix (KAPA Biosystems, Wilmington, MA) was used with Taqman probe (Applied Biosystem) for mouse NADPH oxidase isozymes and mouse GAPDH. KAPA SYBR fast qPCR kit (KAPA Biosystems) was used with the primers listed in Supplementary Table 1. The relative quantitation of gene expression was obtained using the comparative CT method, and the results were normalized by GAPDH or 18 S rRNA as the house-keeping genes. Quantitative PCR analysis was performed in duplicates using an Applied Biosystems 7300 Real-Time PCR System (Applied Biosystems, Forster City, CA).

### Immunoblotting Analyses

Cells were lysed in RIPA buffer (0.1% SDS, 50mM Tris-HCl pH8.0, 150mM NaCl, 0.5mM EDTA, and 1% NP-40) supplemented with a cocktail of protease inhibitors (Sigma). Cellular extracts were quantified for protein using BCA assay kit (Thermo Scientific Pierce, Rockford, IL), subjected to SDS-PAGE and electrotransferred to polyvinylidine difluoride membrane. For immunoblotting assays, anti-Nox4 [20], anti-Oct4 (H-134; Santa Cruz Biotechnology), anti-Sox2 (Millipore), anti-Klf4 (Santa Cruz Biotechnology), anti-c-Myc (Cell Signaling Technology, Danvers, MA), anti-β-Actin (Santa Cruz Biotechnology), anti-p-AKT (Cell Signaling Technology), anti-AKT (Cell Signaling Technology), anti-p-Erk-1,2 (Cell Signaling Technology), anti-Erk-1,2 (Cell Signaling Technology) and anti-α-tubulin (Sigma) antibodies were used. A horseradish peroxidase-conjugated secondary antibody (Invitrogen) was applied prior to visualization by chemiluminescence (Amersham Imager 680; GE Healthcare Life Sciences, Chicago, USA).

### Flow Cytometry

Analyses for apoptosis were performed using FITC Annexin V Apoptosis Detection Kit I (BD Pharmingen, San Diego, CA) according to the manufacturer’s instructions. Cells were analyzed by FACS Calibur (BD Bioscience, San Jose, CA, USA).

### RNA-Sequencing and Bioinformatics Analysis

RNA-seq was performed on the Illumina TruSeq (Stranded mRNA LT Sample Prep Kit) with paired-end reads of 101 bp length. Raw reads were trimmed first by fastx_toolkit (version 0.0.14) to remove low-quality reads and adaptor sequences. After trimming, the reads were mapped to the mouse genome (mm10, GRCm38.97 from Ensembl) using STAR software (version 2.6.0c). Transcript abundance was estimated at the gene level by RSEM version 1.3.1, and gene counts were normalized to the values of Transcript Per Million (TPM) values for the visualization of sample comparisons. 33,860 genes with at least one sample showing nonzero counts were analyzed. The edgeR was used for Exact Test with TMM normalization, to identify differentially expressed genes (DEGs) between wild type (WT) and KO. The EBSeq-HMM was used to identify DEGs with dynamic time patterns in WT and KO, respectively, after the upper quartile normalization. 597 DEGs were selected as a common and specific result of the two tools, EBSeq-HMM and edgeR (FDR<1e-10 and FDR<0.001, respectively). The ConsensusPathDB (http://cpdb.molgen.mpg.de/MCPDB) was used for the over-representative analysis of KEGG and GO (Gene Ontology) Biological_Process with *P*-value≤0.05. All static analysis and visualizations were performed using R version 3.6.1. RNA-seq data sets are available from the GEO database under accession number GSE180912.

## Results

### ROS are Required for Differentiation of EB

**Fig. 1:**
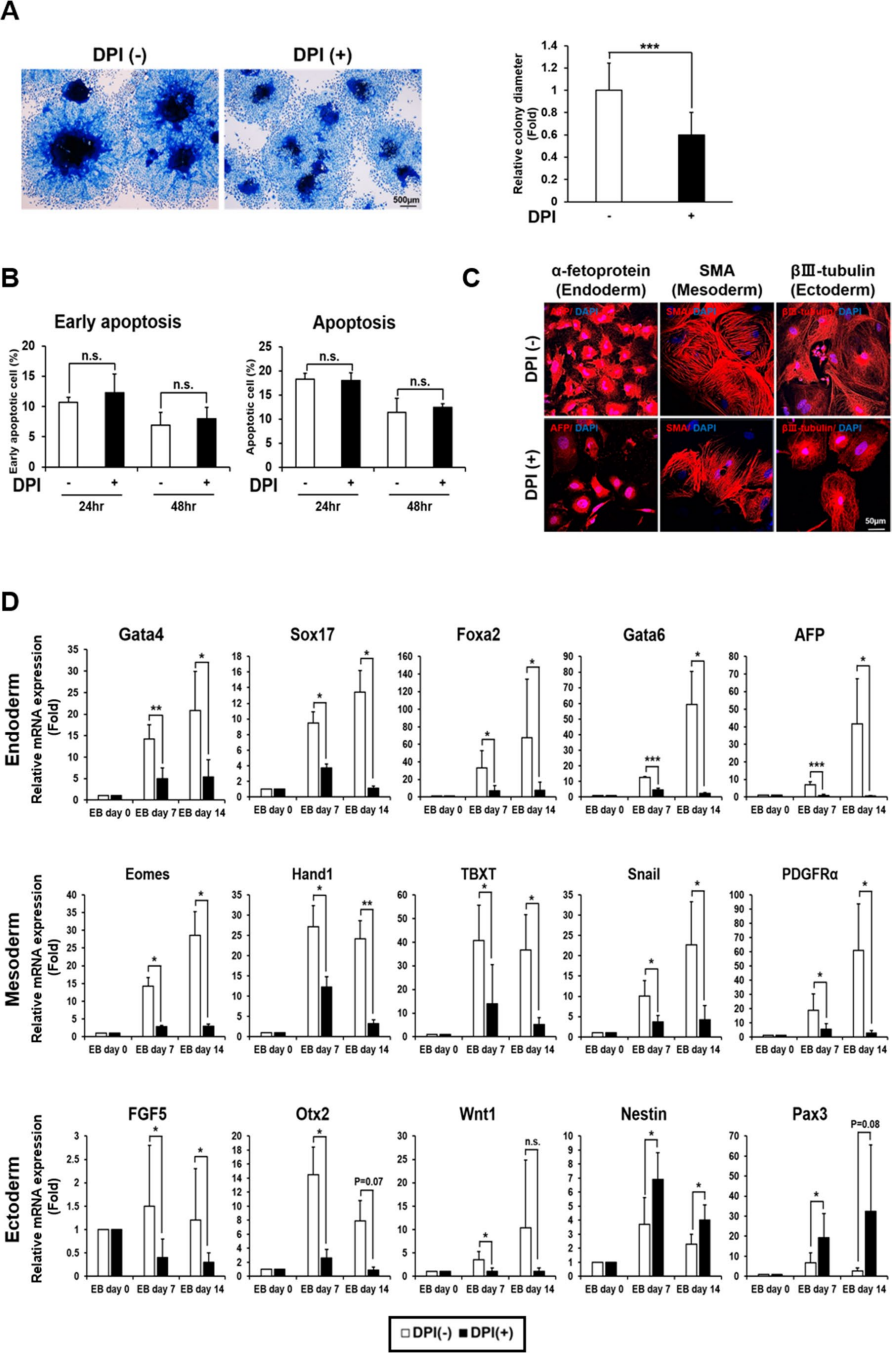
ROS is required for efficient differentiation of EB. (**A**) EBs cultured in differentiation media for 7 days with or without addition of 250 nM DPI and Coomassie blue-stained. Scale bar: 500 μm. Graph on the right side shows diameters of multiple EB colonies from ± DPI cultures with a significant difference (***P-value of <0.0005 from Student’s *t*-test). (**B**) Graph summarizing results from flow cytometric analyses for apoptosis following treatment with DPI for 24 and 48 h. Early apoptosis indicates % Annexin V+ cells, and late apoptosis indicates % PI+ Annexin V+ cells. Note that no significant differences were seen between treated and untreated controls (‘n.s.’ indicates ‘not significant’ from Student’s *t*-test). (**C**) Representative images of immunofluorescence staining showing expression of representative markers of the three embryonic germ layers. βIII-tubulin, SMA, and α-fetoprotein are used as markers for ectoderm, mesoderm, and endoderm respectively. Cells from EBs are cultured with or without 250 nM DPI for 7 days and counterstained with DAPI (blue). Scale bar: 50 μm. (**D**) Real time RT-PCR analyses showing changes in representative marker gene expression during EB differentiation. 5 markers for each of the germ layers are examined on days 0, 7 and 14. Error bars represent mean ± S.D. from five independent experiments. Statistical significance is indicated (*P-value of <0.05, ** *P-*value of <0.005, *** *P-*value of <0.0005 from Student’s *t*-test)

EBs plated on collagen typically generate clearly visible outgrowth of cells after several days in culture. Upon addition of DPI, an inhibitor of Nox enzymes, we saw a significant reduction in the overall size of the outgrowth (Fig. 1A). This difference in colony size was apparently not due to different levels of apoptosis as flow cytometric analyses showed no significant difference between control and DPI-treated cells (Fig. 1B). Interestingly, application of N-acetyl Cysteine (NAC) did not lead to reduction in the size of outgrowth suggesting that while activation of specific signaling by Nox enzyme or enzymes is required for the EB outgrowth, the global removal of ROS mediated by NAC has other benefits for EB growth (Supplementary Fig. 1). Immunostaining for representative lineage markers of the three embryonic germ layers, α-fetoprotein for endoderm, smooth muscle actin (SMA) for mesoderm and βIII-tubulin for ectoderm, indicated that the extent of differentiation was visibly different (Fig. 1C). While terminally differentiated cells expressing specific markers were readily found in clusters in control cultures, the addition of DPI reduced the number of such cells. To quantitatively examine the differentiation status, we examined the expression of multiple marker genes, widely used for confirmation of differentiation into cells of the three germ layers, on days 0, 7 and 14 (Fig. 1D). Treatment of DPI inhibited expression of all tested marker genes for endodermal and mesodermal cells. Interestingly, this was not the case with ectoderm as some marker genes such as FGF5, Otx2 and Wnt1 were inhibited by DPI while others such as Nestin and Pax3 were in fact up-regulated, possibly indicating a more complex response to ROS signaling in the developing ectoderm.

### Nox4 is Required for Efficient EB Differentiation

**Fig. 2:**
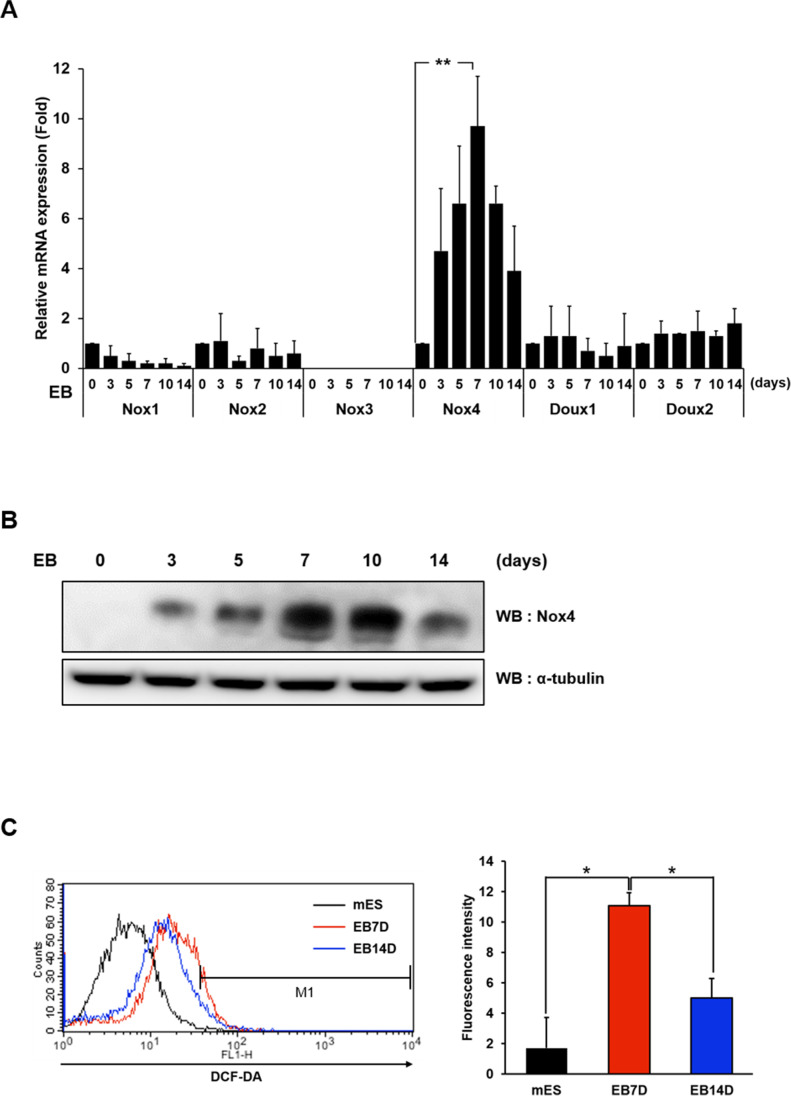
Expression of Nox4 during EB differentiation. (**A**) Expression of Nox isozyme genes during differentiation. Real time RT-PCR analyses on samples from indicated days show only Nox4 is up-regulated peaking on day 7 (**P-value of <0.005 from Student’s *t*-test). (**B**) Immunoblot showing changes in Nox4 expression. α-tubulin is used as the loading control. (**C**) Levels of ROS are examined by DCF-DA fluorescence. Histograms are color-coded for differently for days 0 (mouse embryonic stem cells: mES), 7 and 14 after initiation of differentiation. Graph to the right shows results from two independent flow cytometric analyses. Percentages of cells within the indicated range are shown. Statistical significance is indicated (*P-value of <0.05 from Student’s *t*-test)

We sought to identify the specific Nox isozyme or isozymes responsible for generation of ROS during EB differentiation. To this end, we carried out real time RTPCR analyses using cDNA prepared from various time points during the culture. Importantly, only Nox4 showed a significant up-regulation peaking on day 7 thus indicating that it is the major source of ROS (Fig. 2A ). This was also confirmed at the protein level by immunoblotting for Nox4 (Fig. 2B). Consistently, DCF-DA fluorescence indicated that the ROS level was low at the beginning, peaked around day 7 and declined by day 14 (Fig. 2C).

**Fig. 3:**
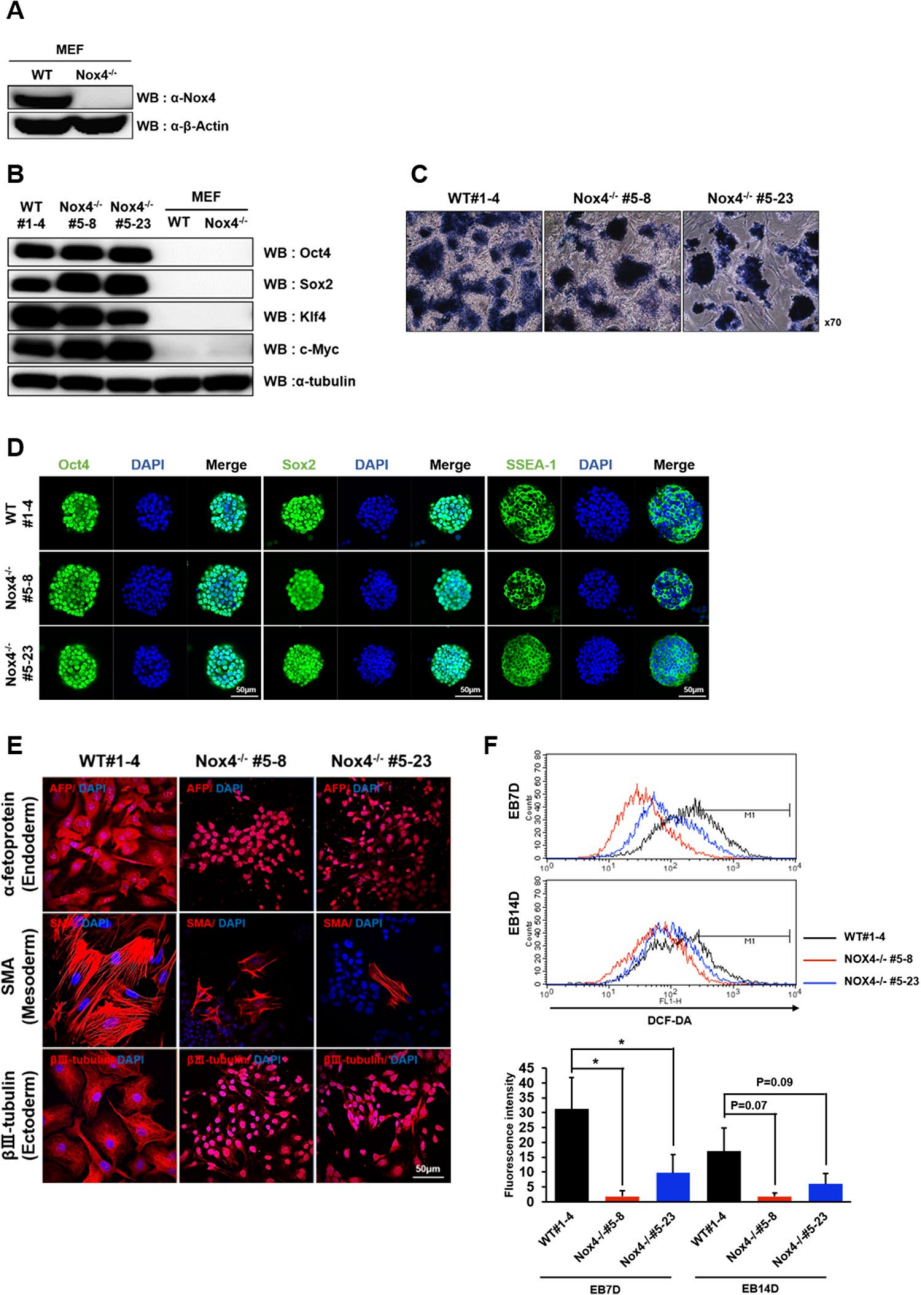
Generation and characterization of Nox4^−/−^ iPSCs. (**A**) Immunoblot showing the absence of Nox4 protein in mouse embryonic fibroblasts (MEF) isolated from Nox4^−/−^ mouse. (**B**) Immunoblot showing expression of Yamanaka factors in iPSCs from wild type (WT) and Nox4^−/−^ mice. MEF are used as negative controls. (**C**) Alkaline phosphatase activity in iPSCs from WT and Nox4^−/−^ mice. (**D**) Immunostaining for expression of Oct4, Sox2 and SSEA-1 in iPSCs from WT and Nox4^−/−^ mice. Scale bar: 50 μm. (**E**) Representative images of immunofluorescence staining showing expression of representative markers of the three embryonic germ layers. Cells from iPSC-derived EBs were cultured, immunostained and counterstained with DAPI (blue). Scale bar: 50 μm. (**F**) Levels of ROS are examined by DCF-DA fluorescence on days 7 and 14 after initiation of differentiation. Histograms are color-coded for different cells under examination. Graphs below show results from three independent flow cytometric analyses. Percentages of cells within the indicated range are shown. Statistical significance is also indicated (*P-value of <0.05 from Student’s *t*-test)

To investigate the role of Nox4 in EB differentiation, we generated iPSCs by introducing Oct4, Sox2, Klf4 and C-Myc genes via viral transduction to embryonic fibroblasts from wild type (WT) and Nox4^−/−^ mice [21] and chose one wild type iPSC line, WT#1-4 and two independent Nox4^−/−^ iPSC lines, Nox4^−/−^ #5-8 and Nox4^−/−^ #5-23 for subsequent analyses (Fig. 3A-B). All three lines of iPSC grew normally and were positive for alkaline phosphatase activity (Fig. 3C). Colonies also expressed Oct4, Sox2 and SSEA-1 indicating that these cells behaved as pluripotent stem cells (Fig. 3D). However, WT cells and Nox4^−/−^ showed dramatically different levels of differentiation upon induction. We examined the extent of expression of the representative markers, α-fetoprotein, SMA, and βIII-tubulin. While these markers were readily seen in clusters of fully differentiated and large-sized cells from WT iPSCs, marker-positive cells from Nox4^−/−^ iPSCs were relatively small in size and found in isolation rather than in clusters indicating that robust differentiation was not taking place (Fig. 3E). We also examined the levels of ROS during differentiation using DCF-DA fluorescence and found consistently that Nox4-/- cells contained significantly lower levels ROS, particularly on day 7 (Fig. 3F).

### RNAseq Analyses for iPSCs

**Fig. 4:**
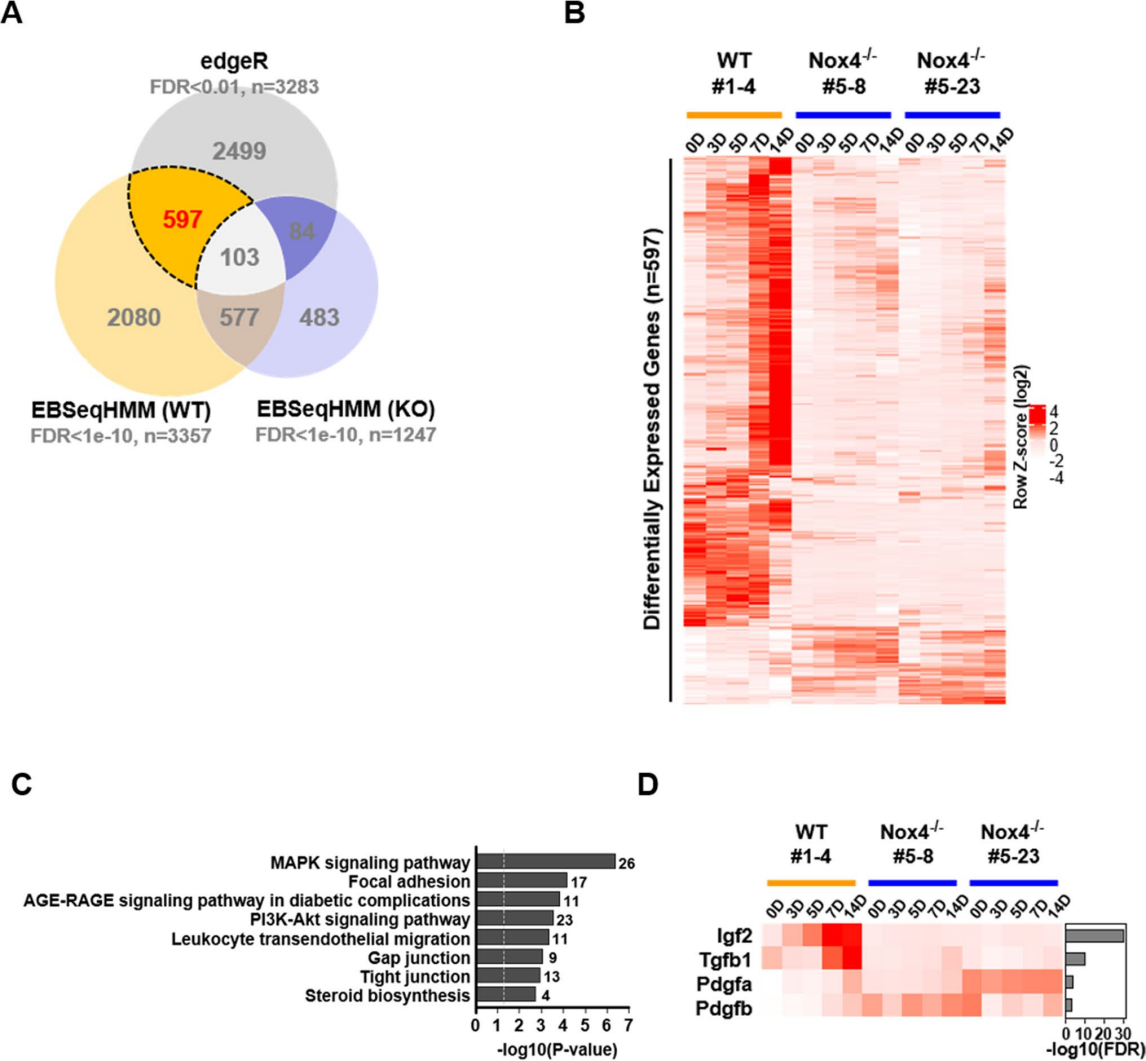
Time-course analysis of transcriptional changes in WT and Nox4^−/−^ EB cells. (**A**) Venn diagram of DEGs (Differentially Expressed Genes) between wild-type and Nox4^−/−^ cells determined by two analytical tools, edgeR and EBSeqHMM. 597 DEGs were selected as genes with significant (FDR <0.01 in edgeR and FDR <1e-10 in EBSeqHMM) patterns in WT compared to Nox4^−/−^ cells. (**B**) Heatmap of log2 TPM (Transcripts Per Million) expression of 597 DEGs in WT and Nox4^−/−^ cells. The heatmap was scaled to row-wise z-score. (**C**) Gene set over-representation analysis of KEGG pathways for 597 DEGs by ConsensusPathDB. Pathways with FDR <0.05 are shown. The full list and details are in the Supplementary Table 2. The number of DEGs in each pathway is indicated next to the bar. The dotted vertical line indicate *P*-value of 0.05. (**D**) Log2 TPM expression for growth factors among DEGs in the MAPK signaling pathway. Genes were sorted by FDR of edgeR shown in the bar plot to the right of the heatmap

Next, we carried out RNAseq analyses to characterize the differences among the iPSC lines at the molecular level. Total RNA preparations were made on 0, 3, 5, 7 and 14 days from EB cultures under differentiation conditions using the three iPSC lines. RNA samples were subjected to mRNA sequencing (RNA-seq), and STAR and RSEM were used for mapping and quantification of RNA-seq data respectively. We identified 3283 genes differentially expressed between WT and Nox4-/- cells by pooling expression data from all time points using edgeR program. We also identified 3357 and 1247 genes that showed significant changes in gene expression over the course of differentiation from WT and Nox4-/- cells respectively using EBSeqHMM program. We focused on genes whose expression levels changed significantly as differentiation proceeded in WT but not in Nox4^−/−^ cells and in the end differed significantly between the two cell groups. These genes, totaling 597, should include down-stream target of Nox4^−/−^ signaling (Fig. 4A, B). Gene set analysis was carried out using ConsensusPathDB application for KEGG pathways and gene ontology (GO) terms [22]. Significant KEGG pathways were diverse as expected for differentiation into multiple lineages (Fig. 4C; Supplementary Table 2). Several of them such as ‘leukocyte transendothelial migration’ and ‘axon guidance’ likely reflected specific cell type differentiation, while others such as ‘focal adhesion’ and ‘gap junction’ indicated changes in the physical nature of cellular environments.

### IGF2 is a Downstream Target of Nox4 Signaling Involved in EB Differentiation

**Fig. 5:**
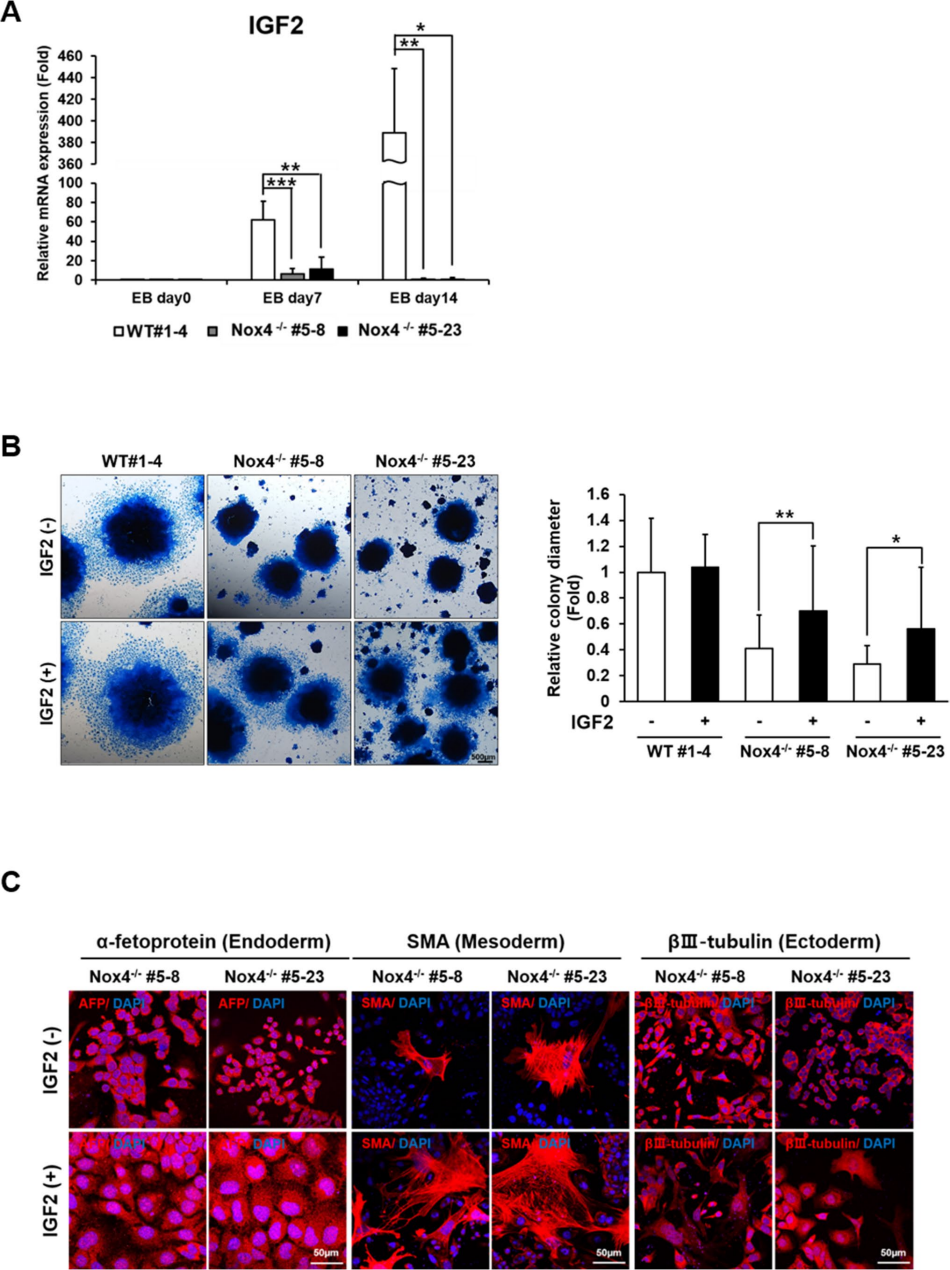
Nox4 promotes differentiation of three embryonic germ layers via IGF2 induction. (**A**) Expression of IGF2 during differentiation of EB cells (*P-value of <0.05, ** *P-*value of <0.005, *** *P-*value of <0.0005 from Student’s *t*-test) from real time RT-PCR analyses. Note the reduced levels in Nox4^−/−^ cells. (**B**) EBs cultured in differentiation media for 7 days with or without addition of IGF2 and Coomassie blue-stained. Graph on the right side shows diameters of multiple EB colonies from ± IGF2 cultures with significant difference (*P-value <0.05 from Student’s *t*-test) in the case of Nox4^−/−^ EBs. (**C**) Representative images of immunofluorescence staining showing expression of representative markers of the three embryonic germ layers. Cells from Nox4^−/−^ EBs are cultured with or without IGF2 for 7 days and counterstained with DAPI (blue). Scale bar: 50 μm

The pathways shown to be most significantly activated in the WT but not in Nox4-/- cells included MAPK and PI3K-Akt signaling pathways. We examined the phosphorylation state of ERK and AKT by immunoblotting on days 0, 7 and 14 and found that the levels of p-AKT and p-ERK were higher in WT cells than in Nox4-/- cells (Supplementary Fig. 2). Notable targets of MAPK pathway included multiple secreted factors among which was IGF2 (Fig. 4D). Most importantly, IGF2 was continuously up-regulated during differentiation of WT EB cells but not during differentiation of Nox4^−/−^ EB cells as also confirmed by real time RTPCR analysis (Figs. 4D and 5A). Importantly, upon addition of IGF2 to differentiating culture, we saw a significant recovery in the size of expanding clones (Fig. 5B). We also examined differentiation markers of the three germ layers and found that a far more robust differentiation took place for iPSCs from both Nox4^−/−^ clonal lines in the presence of IGF2 (Fig. 5C). Finally, we examined the changes in gene expression patterns using multiple markers by RTPCR. Consistent with involvement of IGF2 in differentiation of multiple cell types, we saw significant upregulation in the expression of the majority of tested genes as differentiation progressed (Supplementary Fig. 3A-C).

In sum, we have for the first time shown that Nox4 is the major source of ROS during differentiation of EB into cells of all three germ layers. We also demonstrated that IGF2 is an important downstream effector of Nox4 signaling and mediates induction of multiple genes involved in differentiation. Further elaboration of Nox4-IGF2 axis may provide ways to regulate maintenance and differentiation of pluripotent stem cells.

## Discussion

The notion that reactive oxygen species (ROS) are just cytotoxic reagents is no longer tenable as their role as second messengers is firmly established by extensive body of evidence [1, 2]. For various cell-to-cell signaling events, it is often the group of Nox enzyme complexes that are responsible for receptor-mediated generation of ROS [4, 5]. Regulation by ROS affects critical cellular processes including cell growth, differentiation and apoptosis, and as such expression and activation of Nox enzymes are tightly regulated [4, 23].

Involvement of Nox isozymes in self-renewal and differentiation of various types of stem cells has also been reported [6, 8]. To the best of our knowledge, no study has been carried out analyzing the role of Nox enzyme in differentiation of EB which is not just a three dimensional suspension of cellular aggregate but a complex tissue simulating early embryonic development and containing cells of all three germ layers as well as attendant cell-cell interactions. It was therefore somewhat surprising, given the multitude of lineages differentiating, that only Nox4 was significantly upregulated during the differentiation. We cannot rule out the possibility that for certain specific cell types other Nox isozymes play important roles. Of interest, Nestin and Pax3 did not show compromises in activation in Nox4 -/- EBs. It is possible that these two genes require another Nox enzyme for activation. Alternatively, the lack of down-regulation of the two genes may have to do with the diverse use of ROS signaling in neural development. Specifically, depending on the types of neurons, ROS was shown to have opposite effects in differentiation [24]. At any rate, the overall importance role of Nox4 is clearly demonstrated as EBs lacking this enzyme showed significantly reduced levels of differentiation for all three germ layers as well as overall growth.

Our transcriptomic analyses revealed multitude of signaling pathways and genes that are regulated by Nox4 signaling. We focused on IGF2 which is a component gene of the MAPK signaling pathway as well as of the PI3K-Akt signaling pathway. IGF2 and TGF-β1 to a lesser extent represent growth factors most highly differentially expressed as WT EB differentiation but not Nox4^−/−^ EB differentiation proceeded. IGF2 is known to be highly expressed during embryogenesis and down-regulated in adults except in the brain and intestine [25, 26]. Importantly, IGF2, an autocrine expressed as early as at the two-cell stage functions as an important growth factor for fetal growth [27–30]. Our data in part supports this given that Nox4^−/−^ EB showed developmental lag both in size and in differentiation. Most importantly, the deficiency in differentiation and growth of Nox4 -/- EBs were significantly ameliorated by addition of exogenous IGF2. Clearly, the situation in vivo is much more complex than in EB culture in vitro, and Nox4 affects numerous other genes besides IGF2. Nevertheless, our data for the first time establish the importance of Nox4-IGF2 axis in EB differentiation and possibly in embryogenesis. Members of TGF-β family play diverse and critical roles during embryogenesis [31]. Regulation at least of TGF-β1 by Nox4-mediated signaling is consistent with the significant role of Nox4 and ROS in embryogenesis as well. The exact signaling events that lead to transcriptional activation of IGF and TGF-β1 should be further examined. It is established that Nox4 activates NF-κB and that NF-κB activates TGF-β genes [32–35]. It has also been reported that Nox signaling leads to activation of Smad pathway [33]. Although these observations are often in different cellular contexts, well-established pathways and signaling factors should serve as guides in further detailing of the signaling pathways.

In conclusion, we demonstrate that Nox4 isozyme is responsible for generation of ROS required for efficient differentiation of cells within EB into various types cells found in all three embryonic germ layers. Importantly, we provide evidence indicating that IGF2 is the prime target of Nox4 signaling thereby establishing a basis for elaborating molecular pathways for the control of pluripotent stem cells.

## Supplementary Information

Supplementary Fig. 1 (A) EBs cultured in differentiation media for 7 days with or without addition of 500 nM N-acetyl Cysteine (NAC). Graph on the right side shows diameters of multiple EB colonies from ± NAC cultures without significant difference (‘n.s.’ indicates ‘not significant’ from Student’s *t*-test) (B) Real time RT-PCR analyses showing expression of representative markers during EB differentiation ± NAC. 2 markers for each of the germ layers are examined on days 0 and 7. Error bars represent mean ± S.D. from three independent experiments (‘n.s.’ indicates ‘not significant’ from Student’s *t*-test). (JPG 146 KB)

Supplementary Fig. 2 Immunoblotting analyses for activation of AKT and ERK. Samples are prepared from indicated cells on indicated culture days. α-tubulin is used as the loading control. (JPG 93.3 KB)

Supplementary Fig. 3 (A-C) Effect of IGF2 on expression of representative marker genes was examined by real time RT-PCR using RNA prepared from day 7 EB cultures of the two of Nox4^−/−^ cell lines (*P-value <0.05 and ** *P*-value <0.005 from Student’s *t*-test; ‘n.s.’ indicates ‘not significant’). (JPG 233 KB)

Supplementary Table 1 (DOCX 16.6 KB)

Supplementary Table 2 (DOCX 37.8 KB)

## Data Availability

Data generated during the current study are presented in the article or in the Supplementary Information files. Further details are available upon request. RNA-seq data sets are available from the GEO database under accession number **GSE180912**.

## Abbreviations

EB: embryoid body
iPSCs: induced pluripotent stem cells
IGF2: insulin-like growth factor 2
ES: embryonic stem
E: embryonic day
MEF: mouse embryonic fibroblast
WT: wild type
