# Supplementary figures and images for: Nox4-IGF2 Axis Promotes Differentiation of Embryoid Body Cells Into Derivatives of the Three Embryonic Germ Layers
Source: Stem Cell Rev Rep. 2021 Nov 20;18(3):1181–92. doi: 10.1007/s12015-021-10303-x (PMC8942977; doi:10.1007/s12015-021-10303-x)

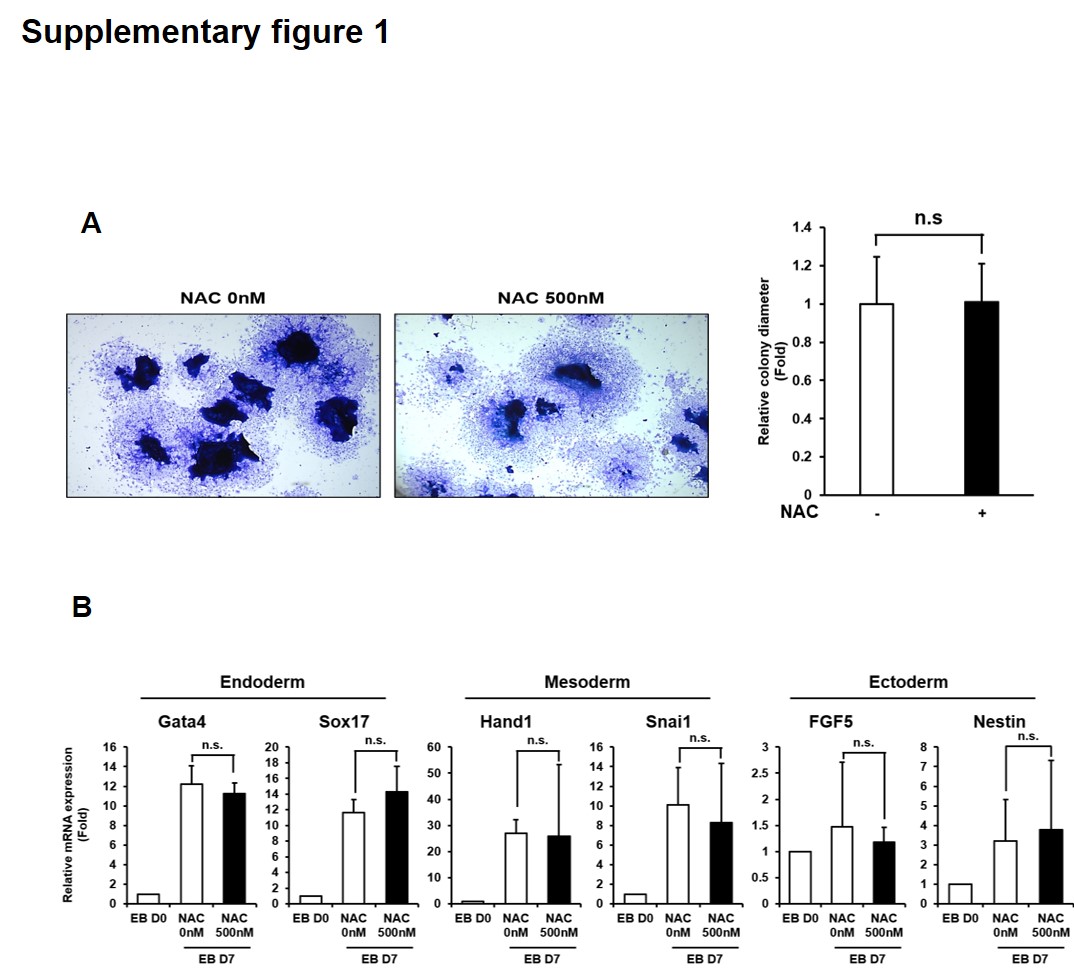

Supplement: Supplementary file 1 — (A) EBs cultured in differentiation media for 7 days with or without addition of 500 nM N-acetyl Cysteine (NAC). Graph on the right side shows diameters of multiple EB colonies from ± NAC cultures without significant difference (‘n.s.’ indicates ‘not significant’ from Student’s t-test) (B) Real time RT-PCR analyses showing expression of representative markers during EB differentiation ± NAC. 2 markers for each of the germ layers are examined on days 0 and 7. Error bars represent mean ± S.D. from three independent experiments (‘n.s.’ indicates ‘not significant’ from Student’s t-test). (JPG 146 KB) [file 12015_2021_10303_MOESM1_ESM.jpg]

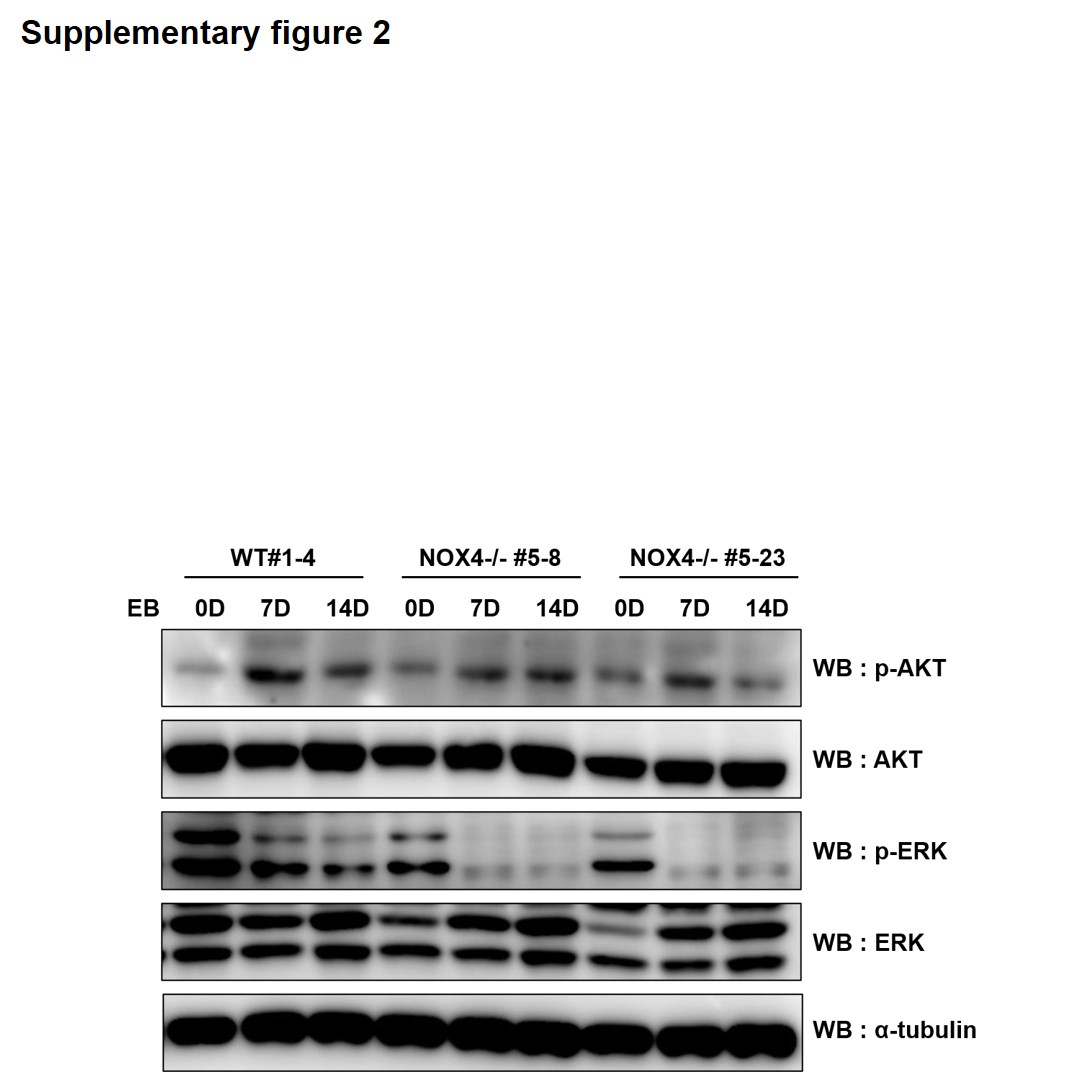

Supplement: Supplementary file 2 — Immunoblotting analyses for activation of AKT and ERK. Samples are prepared from indicated cells on indicated culture days. α-tubulin is used as the loading control. (JPG 93.3 KB) [file 12015_2021_10303_MOESM2_ESM.jpg]

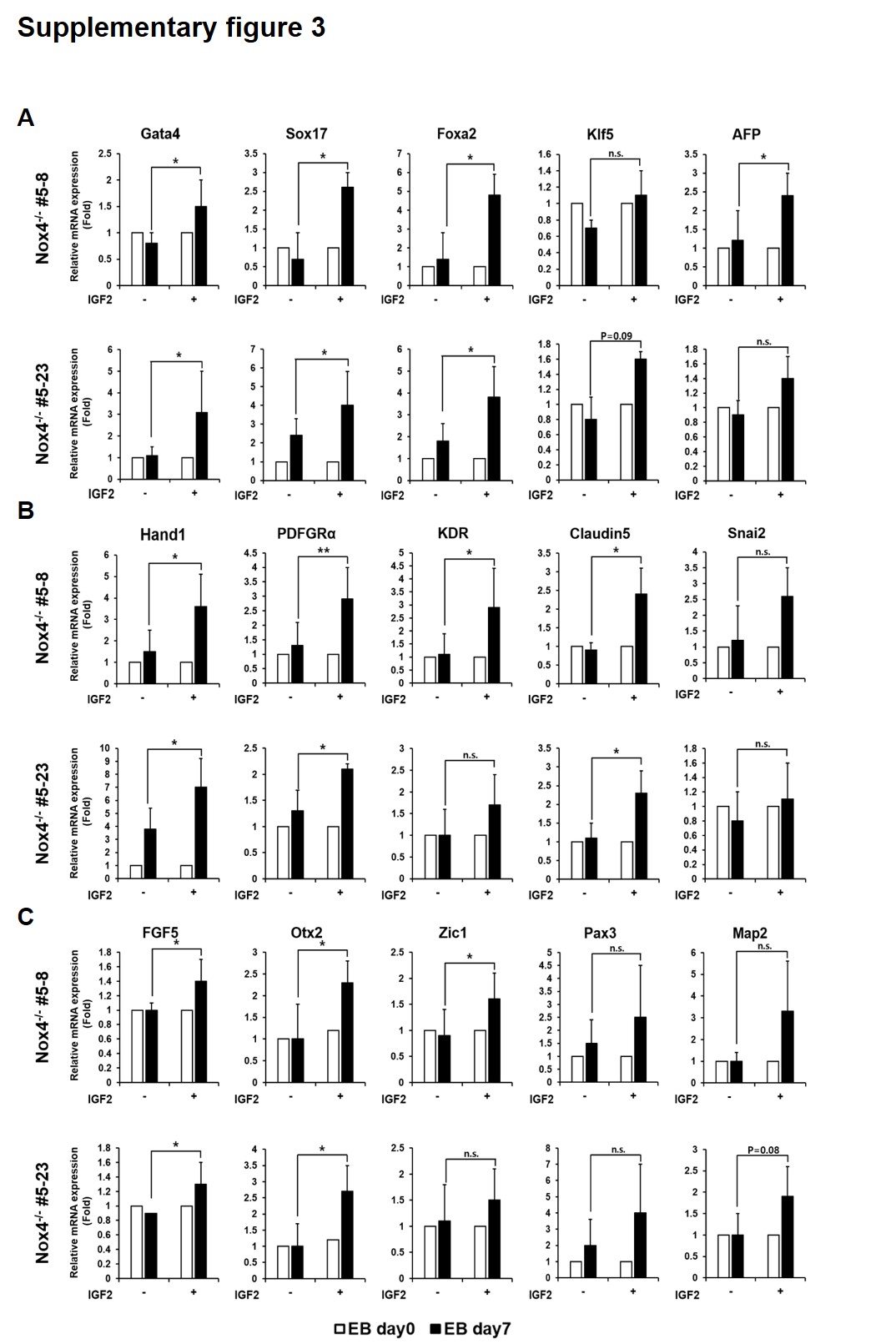

Supplement: Supplementary file 3 — (A-C) Effect of IGF2 on expression of representative marker genes was examined by real time RT-PCR using RNA prepared from day 7 EB cultures of the two of Nox4−/− cell lines (*P-value <0.05 and ** P-value <0.005 from Student’s t-test; ‘n.s.’ indicates ‘not significant’). (JPG 233 KB) [file 12015_2021_10303_MOESM3_ESM.jpg]
