# Supplementary material for: Nox4-IGF2 Axis Promotes Differentiation of Embryoid Body Cells Into Derivatives of the Three Embryonic Germ Layers
Source: Stem Cell Rev Rep. 2021 Nov 20;18(3):1181–92. doi: 10.1007/s12015-021-10303-x (PMC8942977; doi:10.1007/s12015-021-10303-x)
Supplement: Supplementary file 4 — (DOCX 16.6 KB) [file 12015_2021_10303_MOESM4_ESM.docx]

| **Supplementary Table1. Sequences of oligonucleotide primers used in RT-PCR** | | |
| --- | --- | --- |
|  |  |  |
| **Gene** | **Forward primer (5’-3’)** | **Reverse primer (5’-3’)** |
| **GATA binding protein 4**  **(Gata4)** | CCC TAC CCA GCC TAC ATG G | ACA TAT CGA GAT TGG GGT GTC T |
| **SRY-box transcription factor 17 (Sox17)** | CGA GCC AAA GCG GAG TCT C | TGC CAA GGT CAA CGC CTT C |
| **Forkhead box A2 (Foxa2)** | AGC ACC ATT ACG CCT TCA AC | CCT TGA GGT CCA TTT TGT GG |
| **GATA binding protein 6**  **(Gata6)** | TCT ACA CAA GCG ACC ACC TCA G | GCC AGA GCA CAC CAA GAA TCC |
| **Alpha fetoprotein (AFP)** | AGC TCA GCG AGG AGA AAT GGT | GTT CAC AGG GCT TGC TTC ATT C |
| **Kruppel Like Factor 5 (Klf5)** | TGG TTG CAC AAA AGT TTA TAC | GGC TTG GCG CCC GTG TGC TTC C |
| **Eomesodermin (Eomes)** | GCG CAT GTT TCC TTT CTT GAG | GGG GTT GAG TCC GTT TAT GTT |
| **Heart and neural crest**  **derivatatives expressed 1 (Hand1)** | CAC CAC CTA CCA CCG CAG TA | CCT TCT TGG GTC CTG AGC CTT T |
| **T-box transcription factor T (*TBXT*)** | TCT CTG GTC TGT GAG CAA TGG T | TGC GTC AGT GGT GTG TAA TGT G |
| **Zinc finger protein SNAI1 (Snail)** | TCT GAA GAT GCA CAT CCG AAG C | TTG CAG TGG GAG CAG GAG AAT |
| **Zinc finger protein SNAI2 (Snai2)** | TGG TCA AGA AAC ATT TCA ACG CC | GGT GAG GAT CTC TGG TTT TGG TA |
| **Platelet-derived growth factor receptor A (*PDGFRa*)** | TCC TTC TAC CAC CTC AGC GAG | CCG GAT GGT CAC TCT TTA GGA AG |
| **Kinase Insert Domain Receptor (*KDR)*** | TTT GGC AAA TAC AAC CCT TCA GA | GCA GAA GAT ACT GTC ACC ACC |
| **Claudin5 (CLDN5)** | GCA AGG TGT ATG AAT CTG TGC T | GTC AAG GTA ACA AAG AGT GCC A |
| **Fibroblast growth factor 5 (*Fgf5*)** | TGT ACT GCA GAG TGG GCA TC | ACA ATC CCC TGA GAC ACA GC |
| **Orthodenticle homeobox 2 (*Otx2*)** | GCG AAG GGA GAG GAC GAC TTT | CTG CTG TTG GCG GCA CTT AG |
| **Wnt family member 1 (*Wnt1*)** | AGC CCT AGC TGC CAA CAG TA | GGA ATT GCC ATT TGC ACT CT |
| **Neuroectodermal stem cell marker (*Nestin*)** | CCC TGA AGT CGA GGA GCT G | CTG CTG CAC CTC TAA GCG A |
| **Paired box gene 3 (*Pax3*)** | AAC AAG CTG GAG CCA ATC AAC TG | CTG AGG TCT GTG GAC GGT GCT A |
| **Zic family member 1 (*Zic1*)** | CTT TTC CCT GCC CGT TTC | CTC GAA CTC GCA CTT GAA GG |
| **Microtubule-associated protein 2 (*Map2*)** | GCC AGC CTC AGA ACA AAC AG | AAG GTC TTG GGA GGG AAG AAC |
| **Insulin-like growth factor 2 (*Igf2*)** | ACA ACT TCG ATT TGA ACC ACA TTC | GAG AGC TCA AAC CAT GCA AAC T |
| ***18S* ribosomal *RNA* (18s)** | AGG AAT TGA CGG AAG GGC ACC | GTG CAG CCC CGG ACA TCT AAG |
