# Supplementary material for: Nox4-IGF2 Axis Promotes Differentiation of Embryoid Body Cells Into Derivatives of the Three Embryonic Germ Layers
Source: Stem Cell Rev Rep. 2021 Nov 20;18(3):1181–92. doi: 10.1007/s12015-021-10303-x (PMC8942977; doi:10.1007/s12015-021-10303-x)
Supplement: Supplementary file 5 — (DOCX 37.8 KB) [file 12015_2021_10303_MOESM5_ESM.docx]

| **Supplementary Table2. Result of gene set over-representative analysis for KEGG pathways** | | | | | | |
| --- | --- | --- | --- | --- | --- | --- |
|  |  |  |  |  |  |  |
|  | **Pathway** | **DEGs_in_pathway** | **DEGs contained** | **Pathway size** | **p-value** | **q-value** |
| **1** | MAPK signaling pathway | Igf2; Pdgfa; Pdgfb; Cacna1c; Dusp8; Dusp5; Flnc; Dusp1; Tgfb1; Rps6ka6; Mknk2; Jun; Angpt1; Dusp10; Map3k2; Ntrk2; Hspa1b; Hspa1a; Flt1; Erbb3; Pdgfra; Cacna2d1; Efna2; Efna1; Gadd45g; Kdr | 26 | 294 | 4.47E-07 | 8.73E-05 |
| **2** | Focal adhesion | Pdgfa; Pdgfb; Ccnd3; Mylk4; Itga1; Vcl; Itga3; Jun; Lama2; Col4a6; Pdgfra; Tnc; Lamb3; Flnc; Thbs4; Kdr; Flt1 | 17 | 199 | 7.00E-05 | 0.006821258 |
| **3** | AGE-RAGE signaling pathway  in diabetic complications | Tgfb1; F3; Pim1; Cdkn1b; Egr1; Jun; Col4a6; Col3a1; Thbd; Serpine1; Plcb1 | 11 | 100 | 0.000149744 | 0.00973336 |
| **4** | PI3K-Akt signaling pathway | Igf2; Pdgfa; Pdgfb; Ccnd3; Col4a6; Lamb3; Angpt1; Cdkn1a; Il7r; Ghr; Cdkn1b; Thbs4; Lama2; Ntrk2; Tnc; Flt1; Erbb3; Pdgfra; Itga1; Itga3; Efna2; Efna1; Kdr | 23 | 358 | 0.000295386 | 0.01440007 |
| **5** | Leukocyte transendothelial migration | Cldn3; Cldn15; Vcl; Cldn11; Rapgef3; Ocln; Thy1; Gnai1; Cldn4; Cldn5; Cldn7 | 11 | 115 | 0.000474017 | 0.018486645 |
| **6** | Gap junction | Pdgfa; Pdgfb; Tjp1; Plcb1; Map3k2; Pdgfra; Htr2a; Itpr2; Gnai1 | 9 | 86 | 0.000854867 | 0.027783187 |
| **7** | Tight junction | Tjp1; Myh7b; Cldn15; Bves; Jun; Mpp4; Cldn11; Ocln; Myh14; Cldn3; Cldn4; Cldn5; Cldn7 | 13 | 167 | 0.001175736 | 0.032752649 |
| **8** | Steroid biosynthesis | Msmo1; Cyp51; Sqle; Lss | 4 | 19 | 0.001920361 | 0.046808808 |
| **9** | Calcium signaling pathway | Il7r; Pdgfa; Pdgfb; Ghr; Pim1; Cdkn1a; Pdgfra; Lep; Lifr; Cntfr; Ccnd3; Ptpn6 | 12 | 165 | 0.002864793 | 0.058924857 |
| **10** | JAK-STAT signaling pathway | Erbb3; Nos2; Plcb1; Pde1b; Mylk4; Cacna1c; Chrm3; Pdgfra; Chrna7; Htr2a; Oxtr; Itpr2; Htr5b | 13 | 187 | 0.003066773 | 0.058924857 |
| **11** | Amoebiasis | Tgfb1; Nos2; Plcb1; Vcl; Lama2; Col4a6; Col3a1; Lamb3; Serpinb3a | 9 | 106 | 0.003454663 | 0.058924857 |
| **12** | Parathyroid hormone synthesis, secretion and action | Plcb1; Vdr; Cdkn1a; Egr1; Mmp25; Hbegf; Itpr2; Mafb; Gnai1 | 9 | 107 | 0.003919245 | 0.058924857 |
| **13** | Protein digestion and absorption | Col15a1; Eln; Col11a1; Col11a2; Col22a1; Col4a6; Col3a1; Dpp4 | 8 | 90 | 0.004584972 | 0.058924857 |
| **14** | Oxytocin signaling pathway | Plcb1; Nfatc2; Nfatc4; Cdkn1a; Cacna1c; Jun; Oxtr; Itpr2; Cacna2d1; Mylk4; Gnai1 | 11 | 153 | 0.005050064 | 0.058924857 |
| **15** | Axon guidance | Nfatc2; Ephb1; Nfatc4; Sema5b; Epha8; Unc5c; Efna2; Epha1; Rnd1; Efna1; Ntn1; Gnai1 | 12 | 175 | 0.00508071 | 0.058924857 |
| **16** | Serotonergic synapse | Plcb1; Htr5b; Alox8; Cacna1c; Alox15; Htr2a; Rapgef3; Itpr2; Dusp1; Gnai1 | 10 | 132 | 0.005119099 | 0.058924857 |
| **17** | Small cell lung cancer | Nos2; Cdkn1b; Cdkn1a; Itga3; Lama2; Gadd45g; Col4a6; Lamb3 | 8 | 92 | 0.00523804 | 0.058924857 |
| **18** | Gastric acid secretion | Plcb1; Mylk4; Chrm3; Sstr2; Slc26a7; Itpr2; Gnai1 | 7 | 74 | 0.005584195 | 0.058924857 |
| **19** | Ferroptosis | Slc39a8; Alox15; Hmox1; Slc7a11; Acsl4 | 5 | 41 | 0.005741396 | 0.058924857 |
| **20** | Cell adhesion molecules (CAMs) | Cldn11; Cldn15; Nrcam; Cd80; Sdc3; Cldn5; Ocln; Cldn3; Cldn4; Cadm3; Cldn7 | 11 | 169 | 0.007686625 | 0.074944596 |
| **21** | Fluid shear stress and atherosclerosis | Pdgfa; Pdgfb; Gstt1; Gsta4; Hmox1; Jun; Nqo1; Thbd; Dusp1; Kdr | 10 | 143 | 0.00886711 | 0.08233745 |
| **22** | ECM-receptor interaction | Lama2; Itga1; Thbs4; Itga3; Col4a6; Tnc; Lamb3 | 7 | 83 | 0.010332699 | 0.091585286 |
| **23** | HIF-1 signaling pathway | Nos2; Mknk2; Cdkn1b; Cdkn1a; Hmox1; Angpt1; Serpine1; Flt1 | 8 | 105 | 0.011361096 | 0.096322333 |
| **24** | Cocaine addiction | Fosb; Gpsm1; Jun; Th; Gnai1 | 5 | 48 | 0.012378637 | 0.100576427 |
| **25** | Toxoplasmosis | Tgfb1; Nos2; Lama2; Ldlr; Hspa1b; Hspa1a; Lamb3; Gnai1 | 8 | 108 | 0.013317719 | 0.100984758 |
| **26** | Cortisol synthesis and secretion | Plcb1; Cyp11a1; Cacna1c; Ldlr; Itpr2; Pde8a | 6 | 68 | 0.013903029 | 0.100984758 |
| **27** | Complement and coagulation cascades | F3; C1s1; Plau; A2m; Serping1; Thbd; Serpine1 | 7 | 88 | 0.013982505 | 0.100984758 |
| **28** | Estrogen signaling pathway | Plcb1; Hbegf; Jun; Hspa1b; Hspa1a; Krt12; Itpr2; Gnai1; Krt18 | 9 | 133 | 0.015495594 | 0.103549944 |
| **29** | GnRH signaling pathway | Plcb1; Egr1; Map3k2; Cacna1c; Jun; Hbegf; Itpr2 | 7 | 90 | 0.015672919 | 0.103549944 |
| **30** | Proteoglycans in cancer | Igf2; Erbb3; Tgfb1; Wnt2; Cdkn1a; Plau; Hbegf; Gpc3; Itpr2; Flnc; Ptpn6; Kdr | 12 | 204 | 0.016106824 | 0.103549944 |
| **31** | Cushing syndrome | Plcb1; Aqp1; Pde1b; Cacna1c; Itpr2; Gnai1 | 6 | 72 | 0.016930824 | 0.103549944 |
| **32** | Renin secretion | Plcb1; Cyp11a1; Cdkn1b; Cdkn1a; Cacna1c; Ldlr; Wnt2; Itpr2; Pde8a; Gnai1 | 10 | 158 | 0.016992811 | 0.103549944 |
| **33** | Hepatitis C | Cldn11; Cldn15; Cdkn1a; Ldlr; Oas2; Ocln; Cldn3; Cldn4; Cldn5; Cldn7 | 10 | 160 | 0.018393499 | 0.108688859 |
| **34** | Apelin signaling pathway | Acta2; Plcb1; Nos2; Ccn2; Mylk4; Egr1; Itpr2; Serpine1; Gnai1 | 9 | 139 | 0.020091605 | 0.113986711 |
| **35** | Pathways in cancer | Blvrb; Enpp1 | 2 | 8 | 0.020981964 | 0.113986711 |
| **36** | Riboflavin metabolism | Igf2; Pdgfa; Pdgfb; Pim1; Ccnd3; Hmox1; Nos2; Tgfb1; Jun; Col4a6; Lamb3; Gnai1; Il7r; Gstt1; Cdkn1b; Cdkn1a; Lama2; Nqo1; Plcb1; Pdgfra; Gsta4; Wnt2; Itga3; Gadd45g | 24 | 530 | 0.0210437 | 0.113986711 |
| **37** | Proximal tubule bicarbonate reclamation | Slc4a4; Car4; Aqp1 | 3 | 22 | 0.024858671 | 0.131011916 |
| **38** | Hypertrophic cardiomyopathy (HCM) | Tgfb1; Lama2; Cacna1c; Itga1; Itga3; Cacna2d1 | 6 | 84 | 0.035257516 | 0.180926724 |
| **39** | Relaxin signaling pathway | Acta2; Plcb1; Nos2; Tgfb1; Jun; Col4a6; Col3a1; Gnai1 | 8 | 131 | 0.037168774 | 0.185843871 |
| **40** | Rap1 signaling pathway | Plcb1; Pdgfb; Pdgfa; Gnai1; Pdgfra; Efna1; Efna2; Rapgef3; Angpt1; Kdr; Flt1 | 11 | 209 | 0.041761224 | 0.20336863 |
| **41** | Dilated cardiomyopathy (DCM) | Tgfb1; Lama2; Itga3; Itga1; Cacna1c; Cacna2d1 | 6 | 89 | 0.042759558 | 0.20336863 |
| **42** | Long-term potentiation | Itpr2; Plcb1; Cacna1c; Rps6ka6; Rapgef3 | 5 | 67 | 0.04489065 | 0.208420877 |
